# Renal tubular epithelial-derived follistatin-like 1 protects against UUO-induced renal fibrosis in mice via inhibiting NF-κB-mediated epithelial inflammation

**DOI:** 10.7150/thno.100969

**Published:** 2025-01-20

**Authors:** Zhuan Niu, Jiasen Guo, Xingzu Liu, Mo Chen, Yueyue Jin, Maolin Yao, Xiaoxu Li, Qianqian Che, Shuzi Li, Chenjie Zhang, Kunyue Shangguan, Dekun Wang, Chuan'ai Chen, Wenli Yu, Xiaoyue Tan, Wen Ning, Lian Li

**Affiliations:** 1 College of Life Sciences, State Key Laboratory of Medicinal Chemical Biology, Nankai University, Tianjin 300071, China.; 2 Department of Pathology, School of Medicine, Nankai University, Tianjin 300071, China.; 3 Department of Anesthesiology, Tianjin First Central Hospital, Tianjin 300192, China.

**Keywords:** FSTL1, renal fibrosis, UUO, renal tubular epithelial inflammation

## Abstract

**Rationale:** Renal fibrosis is commonly recognized as the ultimate pathway for most chronic kidney diseases (CKD). Renal tubular epithelial inflammation drives the initiation and progression of renal fibrosis. Follistatin-like 1 (FSTL1) is a small matricellular protein, whose expression pattern, function and underlying mechanism in regulating renal inflammation and fibrosis remains largely unknown.

**Methods:** We utilized two *Fstl1*-deficient genetic mouse models: heterozygous *Fstl1^+/-^* mice and whole-body *Fstl1* conditional knockout mice, and a mouse model with FSTL1 overexpression via adenoviral vector infection. These mice were subjected to unilateral ureteral obstruction (UUO). We used an *Fstl1* lineage tracing mouse to investigate the expression and location of induced FSTL1 in the obstructed kidney. We investigated the effect of FSTL1 on TNF-α induced epithelial inflammation and the NF-κB pathway by overexpression or knockdown of FSTL1 in human kidney epithelial cells (HK2).

**Results:** We observed increased expression of FSTL1 in kidneys from patients with CKD, and UUO mouse model of renal injury and fibrosis. Deletion of *Fstl1* in mice aggravated UUO-induced inflammatory kidney injury and subsequent fibrosis. Conversely, overexpression of FSTL1 by adenoviral vector infection in mice mitigated expression of proinflammatory cytokines and the fibrotic phenotype. Mechanistically, we identified that increased FSTL1 was mostly derived from the tubular epithelium of the obstructed mouse kidney. FSTL1 inhibited human renal epithelial cell inflammatory responses, as assessed by reducing the NF-κB pathway, release of IL-1β and IL-6, expression of intercellular adhesion molecule 1 (ICAM-1), and monocyte adhesion to kidney epithelial cells.

**Conclusions:** These findings suggest that FSTL1 plays a protective role against kidney fibrosis by inhibiting renal epithelial inflammation via the NF-κB pathway in epithelium, thereby offering a potential novel strategy for treating progressive CKD.

## Introduction

The incidence of chronic kidney disease (CKD) is on the rise in recent years and remains a global health problem 1. During the epidemic of COVID-19, viral infection has been associated with the exacerbation of CKD and substantially elevates the mortality risk of CKD patients 2-4. Renal fibrosis, characterized by excessive extracellular matrix (ECM) deposition, is the final pathological feature of almost all progressive CKD regardless of the underlying etiology 5. Though considerable effort has been dedicated, few strategies are available that completely cure renal fibrosis 6. Therefore, a thorough molecular insight into renal fibrosis is crucial to develop novel therapeutic approaches for the treatment of patients with progressive CKD.

Accumulating evidence indicates that nonresolving inflammation following a sustained injury is pivotal in both the initiation and progression of renal fibrosis 7-9. Anti-inflammatory interventions have shown efficacy in mitigating renal fibrosis 10.

During the inflammatory process, the injured renal tubular epithelial cells are activated and release proinflammatory cytokines, such as IL-1β and IL-6, which are important in initiating the reparative process 9. These activated cells also increase the expression of ICAM-1 (intercellular adhesion molecule 1) on their surface, facilitating the adhesion and migration of monocytes into the renal interstitium 11. Within the post-injury inflammatory milieu, the secretion of pro-fibrotic factors such as TGF-β1 is increased, leading to fibroblast activation, excessive ECM production, and ultimately the progression of kidney fibrosis 9.

Follistatin-like 1 (FSTL1) is widely known as a small matricellular protein in the SPARC family, whose amino acid sequence is highly conserved among species 12. The growing literature has investigated the roles of FSTL1 in development and multiple diseases 13, including cardiovascular disease 14, 15, cancer 16, 17, inflammation and autoimmunity disease 18, 19. We have previously shown that FSTL1 is a mesenchymal-derived matricellular protein that is upregulated in lungs from patients of IPF and in bleomycin model of lung fibrosis 20. We have identified FSTL1 as a novel profibrotic factor for those fibrotic interstitial lung disease (ILD) 20-22. Furthermore, the profibrotic role of FSTL1 has been demonstrated in other organ fibrosis, including heart 15, 23, liver 24 and skin 25. However, the role of FSTL1 in renal fibrosis remains inconsistent. Adams *et al.* reported a role for FSTL1-mediated IL-1β suppression in the protection of the kidney from cisplatin-induced injury using a hypomorphic *Fstl1* genetrap strain 26. Hayakawa *et al.* also showed that FSTL1 mitigates renal injury/fibrosis in a subtotal nephrectomy mouse model using a cardiac-specific *Fstl1*-deficient mouse strain, partly by dampening AMPK activation and inflammatory cytokines in mesangial cells 27. On the other hand, both Maksimowski *et al.* and Zhang* et al.* conducted the single-cell RNA-Seq analysis and localized FSTL1 to interstitial fibroblasts/myofibroblasts 28. Zhang *et al.* further reported that FSTL1 overexpression via hydrodynamic gene delivery exacerbated renal fibrosis in a unilateral ureteral obstruction (UUO) mouse model, through activation of fibroblast via regulating Wnt/β-catenin signaling 29. Accordingly, there has been a call to leverage believable mouse genetic techniques and systems biology approaches to elucidate the role of FSTL1 in renal injury and fibrosis.

Here, we utilized two *Fstl1*-deficient genetic models to confirm the anti-inflammatory and anti-fibrotic role of FSTL1 in UUO-induced kidney fibrosis. We employed an *Fstl1* lineage tracing mouse to show that increased FSTL1 was mainly derived from the tubular epithelium of obstructed mouse kidney. In human tubular epithelial cells, FSTL1 can attenuate NF-κB-mediated inflammatory cytokine secretion and leukocyte infiltration. This study further provides evidence that upregulation of FSTL1 may be beneficial in the treatment of renal fibrosis in which inflammation plays a central role.

## Results

### FSTL1 expression is upregulated in kidneys of CKD patients and UUO-injured mice

**Figure 1:**
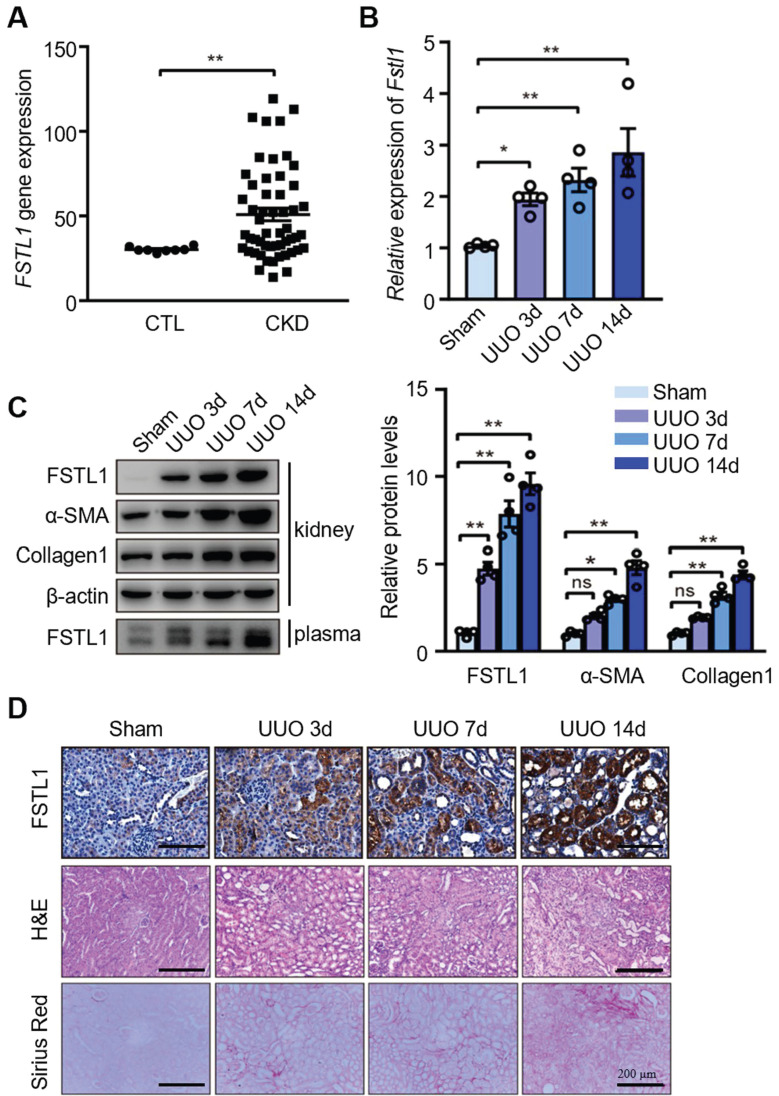
**Increased expression of FSTL1 in the kidneys of CKD patients and in UUO-injured mice.** (A) The expression of FSTL1 in CKD renal tissues was investigated using a previously published gene-profiling dataset specific to CKD kidneys. This involved a microarray analysis comparing *FSTL1* mRNA levels in kidney samples from CKD patients with those from control subjects. (B) qRT-PCR was employed to assess the mRNA levels of *Fstl1* in kidney tissues of C57BL/6J mice at the indicated time points post-UUO surgery, using β-actin as an internal standard for normalization. (C) Western blot analysis was conducted to quantify the protein levels of FSTL1 and fibrosis-associated markers (α-SMA and Collagen 1) in the renal tissues of C57BL/6J mice at the indicated time points following UUO surgery, with the protein levels being normalized to β-actin. The levels of FSTL1 in the plasma from UUO-injured mice were also analyzed using Western blotting. (D) Histological examinations, including H&E staining, Sirius Red staining, and IHC analysis, were performed to visualize FSTL1 in renal sections at predetermined time points after UUO, with representative images being displayed. (A-C) Error bars represent the mean ± standard error of the mean (SEM). Statistical significance was denoted as *P < 0.05, **P < 0.01; ns, not significant.

We first investigated whether FSTL1 expression is aberrant in patients with CKD. We analyzed FSTL1 expression in a gene-profiling dataset of CKD kidneys from a previous study 30 and identified a significant elevation of *FSTL1* mRNA level in CKD kidney tissues compared with control subjects (Figure 1A). Next, through qRT-PCR and western blot analyses, we found that both mRNA and protein expression of FSTL1, as well as circulating levels of FSTL1, were significantly increased in UUO-injured kidneys or plasma in a time-dependent manner, compared to sham-operated kidneys (Figure 1B-C). The increased FSTL1 expression was consistent with the elevated levels of fibrosis-related proteins, including α-SMA and fibronectin (Figure 1C). Through immunohistochemistry, H&E, and sirius red staining, we also observed a marked increase of FSTL1 concurrent with the progression of renal fibrosis induced by UUO (Figure 1D). These findings indicate that FSTL1 is a renal injury-related gene and may contribute to the pathogenesis of kidney interstitial fibrosis.

### FSTL1 deficiency in mice exacerbates renal interstitial fibrosis after UUO

**Figure 2:**
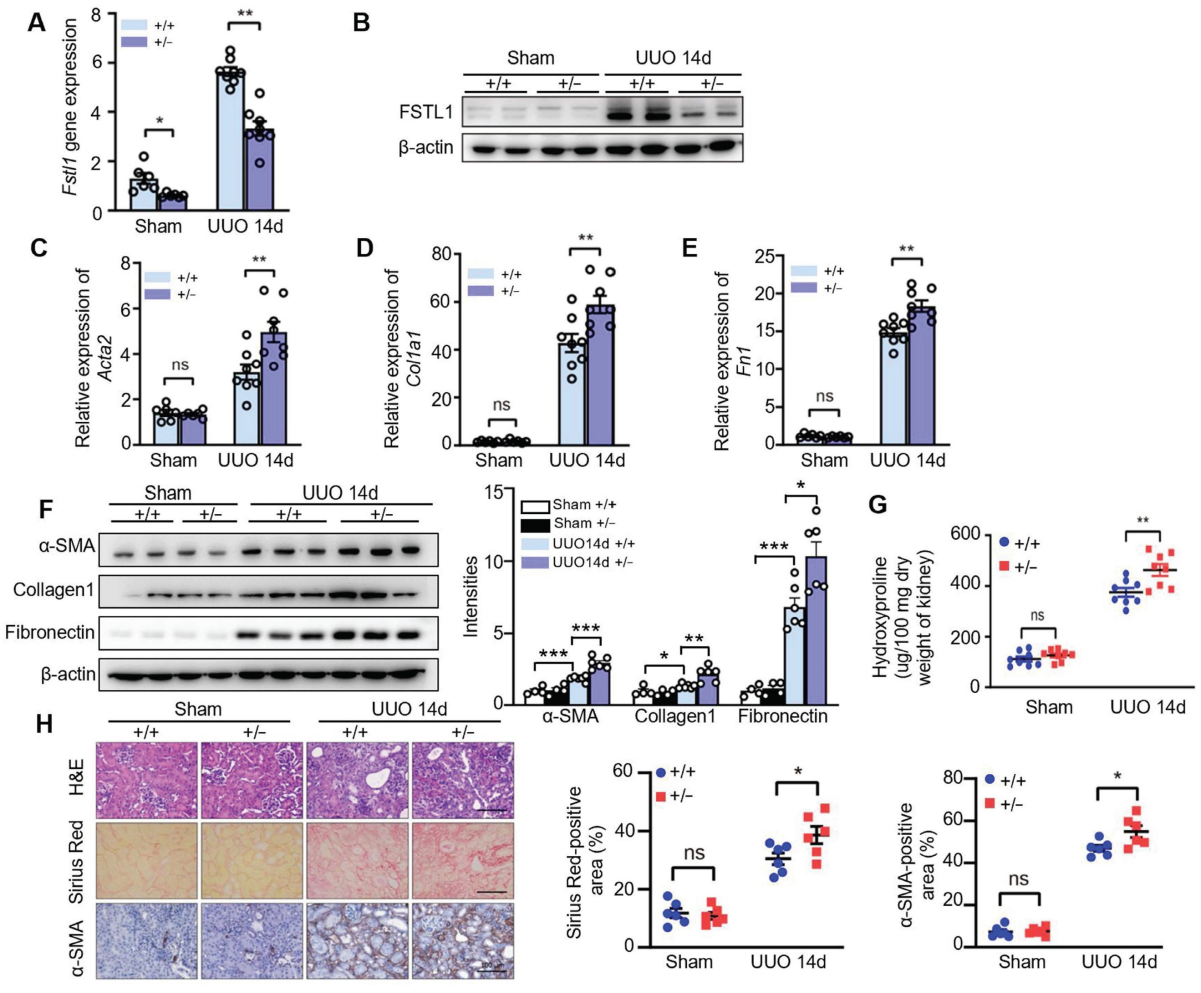
***Fstl1* haplodeficiency aggravates renal interstitial fibrosis after UUO treatment.** *Fstl1^+/-^* and their WT littermates were subjected to UUO surgery, and kidneys harvested at day 14 post-operation were analyzed. (A and B) The expression of FSTL1 in the kidneys, both sham and obstructive, of *Fstl1^+/-^* and WT mice, was assessed using qRT-PCR and western blot analysis. (C-F) Similarly, the expression levels of fibrotic markers α-SMA, Collagen 1, and Fibronectin in sham and obstructive kidney lysates from both WT and* Fstl1^+/-^* mice were evaluated through qRT-PCR (C-E) and western blot analyses (F). Band densitometry was performed using ImageJ. (G) The hydroxyproline content, indicative of collagen deposition, was quantified in the kidney lysates. (H) Histological assessments including H&E staining, Sirius Red staining, and α-SMA immunostaining were performed on sections from sham and UUO kidneys, accompanied by quantitative analyses. (A, C, D and E) The data are normalized to β-actin. (A-H) Error bars represent the mean ± SEM, with statistical significance indicated by *P < 0.05, **P < 0.01.

To investigate the biological significance of the inducible expression of FSTL1 during fibrogenesis, we examined the fibrotic response to UUO-induced kidney injury in *Fstl1*-deficient mice. After 14 days of UUO, there was a notable reduction in both mRNA and protein levels of FSTL1 in the kidneys of *Fstl1^+/-^* mice compared with WT mice (Figure 2A-B). Importantly, *Fstl1^+/-^* mice exhibited more severe renal fibrosis. The mRNA and protein levels of fibrosis-related markers, including α-SMA, collagen 1, and fibronectin were elevated in the obstructed kidneys of *Fstl1^+/-^* mice compared with WT mice (Figure 2C-F). The exaggerated collagen accumulation in the obstructed kidneys of *Fstl1^+/-^* mice was further supported, as assessed by increased hydroxyproline content (Figure 2G) and sirius red staining (Figure 2H). Haplodeletion of *Fstl1* showed a significant increase in inflammatory cell infiltration and renal architectural damage 14 days post-UUO as indicated by H&E staining (Figure 2H). Excessive active fibroblasts in the UUO kidneys of *Fstl1^+/-^* mice were verified by increased α-SMA immunostaining (Figure 2H).

Since the homozygous *Fstl1* mice died shortly after birth, we also generated global depletion of *Fstl1* knockout mice in adulthood using *Ubc-CreER^T2^* (*Ubc-CreER^T2^;Fstl1^flox/flox^*, *Fstl1cKO*) to ascertain the effect of *Fstl1* deletion on UUO-induced renal injury. As shown in Figure S1A-B, *Fstl1* knockout significantly promoted an increase in fibrosis-related markers (α-SMA and fibronectin). Collectively, these *in vivo* data indicate that FSTL1 is induced in response to renal injury and acts as an anti-fibrotic factor in renal fibrogenesis after UUO.

### FSTL1 deficiency in mice exhibits aggravated inflammatory response after UUO

**Figure 3:**
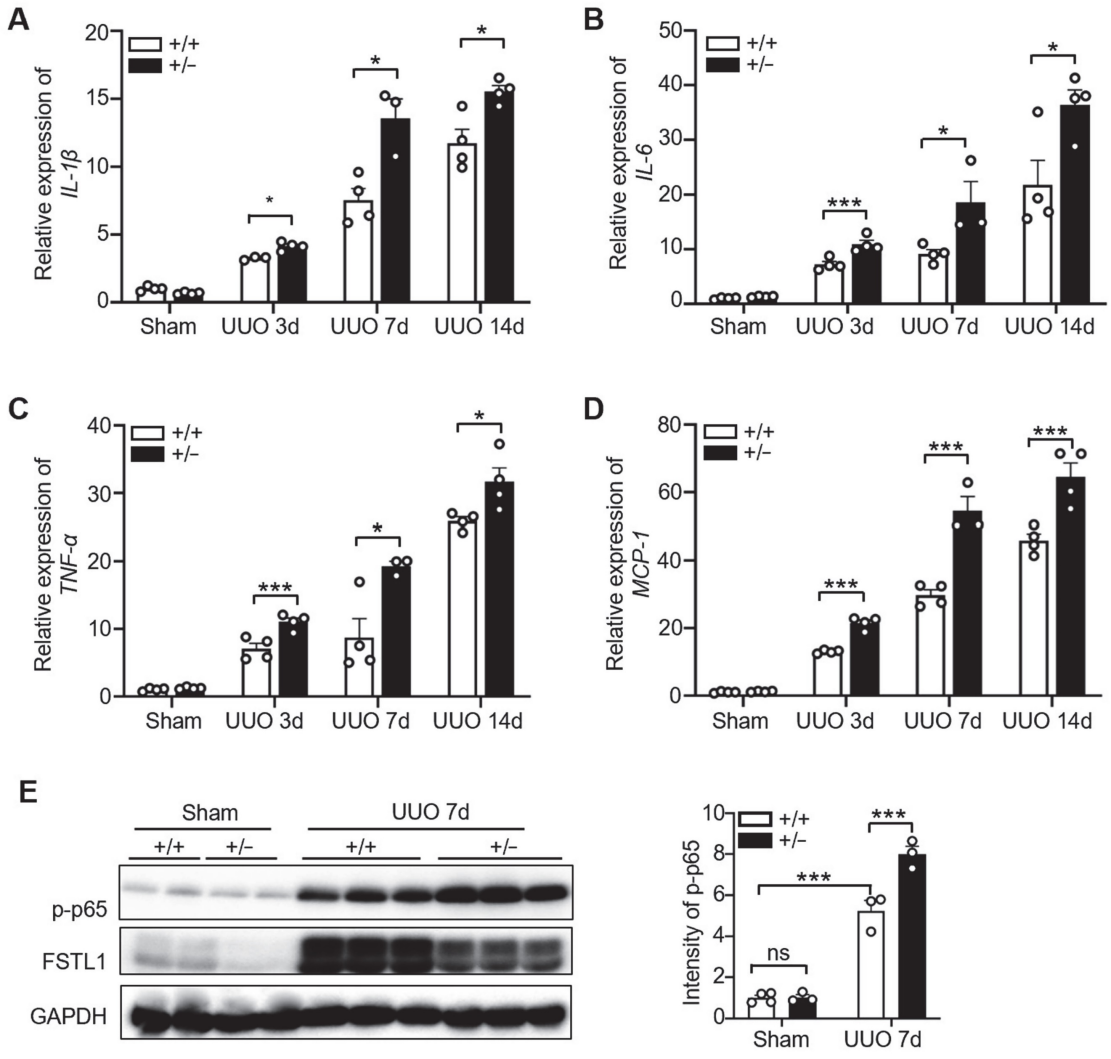
***Fstl1* haplodeficiency promoted UUO-induced kidney inflammation.** (A-D) qRT-PCR was utilized to evaluate the mRNA levels of *IL-1β*, *IL-6*, *TNF-α*, and *MCP-1* in both sham-operated and obstructive kidney lysates from WT and *Fstl1^+/-^* mice after UUO for 3, 7 and 14 days. The quantification of results was normalized to Gapdh. (E) Western blot analysis was performed to assess the levels of phosphorylated p65 (p-p65) in both sham-operated and obstructed kidney lysates from wild-type (WT) and* Fstl1^+/-^* mice after 7 days of UUO (n = 3 per group). Error bars represent the mean ±SEM, with statistical significance denoted by *P < 0.05, **P < 0.01, ***P < 0.001; ns, not significant.

Unresolved inflammation is one of the most important initiators for renal interstitial fibrosis 7, 9. FSTL1 has been recognized as a key inflammatory protein in various diseases 18. Here, we explored the role of FSTL1 in the inflammatory response to UUO-induced kidney injury. We found that obstructed kidneys subjected to 3, 7 and 14 days of UUO exhibited a significant increased expression of proinflammatory cytokines, including* IL-1β*, *IL-6*, *TNF-α*, and *MCP-1*, compared with the sham-operated kidneys (Figure 3A-D). Notably, the obstructed kidneys of *Fstl1^+/-^*mice displayed higher expression of these proinflammatory cytokines compared with those of WT controls (Figure 3A-D).

Activation of the NF-κB pathway triggers the renal inflammatory response 7. Furthermore, we examined the activation of NF-κB pathway in both sham-operated and obstructed kidney lysates from WT and* Fstl1^+/-^* mice. We found that obstructed kidneys subjected to 3 or 7 days of UUO exhibited a significantly increased expression of phosphorylated NF-κB p65 (p-p65) compared with the sham-operated kidneys (Figure 3E and Figure S2A). There is no obvious difference in p-p65 expression between the sham-operated kidneys of WT and *Fstl1^+/-^* mice (Figure 3E). However, the protein levels of p-p65 were significantly higher in the kidneys of *Fstl1^+/-^* mice than in WT mice under UUO injury for 3 (Figure S2A) or 7 days (Figure 3E). These findings suggest that deletion of *Fstl1* in mice exacerbates the UUO-induced the inflammatory response and the activation of NF-κB pathway, which may aggravate UUO-induced renal interstitial fibrosis.

The Wnt/β-catenin pathway has been implicated in kidney fibrosis 29. Here, we performed qRT-PCR analysis and found that obstructed kidneys subjected to 3, 7 or14 days of UUO exhibited a significantly increased mRNA expression of *β-catenin* compared with the sham-operated kidneys (Figure S2B). However, no significant difference in the mRNA levels of β-catenin was observed between the sham-operated and obstructed kidneys of WT and *Fstl1^+/-^* mice (Figure S2B).

### Increased FSTL1 mainly derives from renal tubular epithelium of obstructed mouse kidney

**Figure 4:**
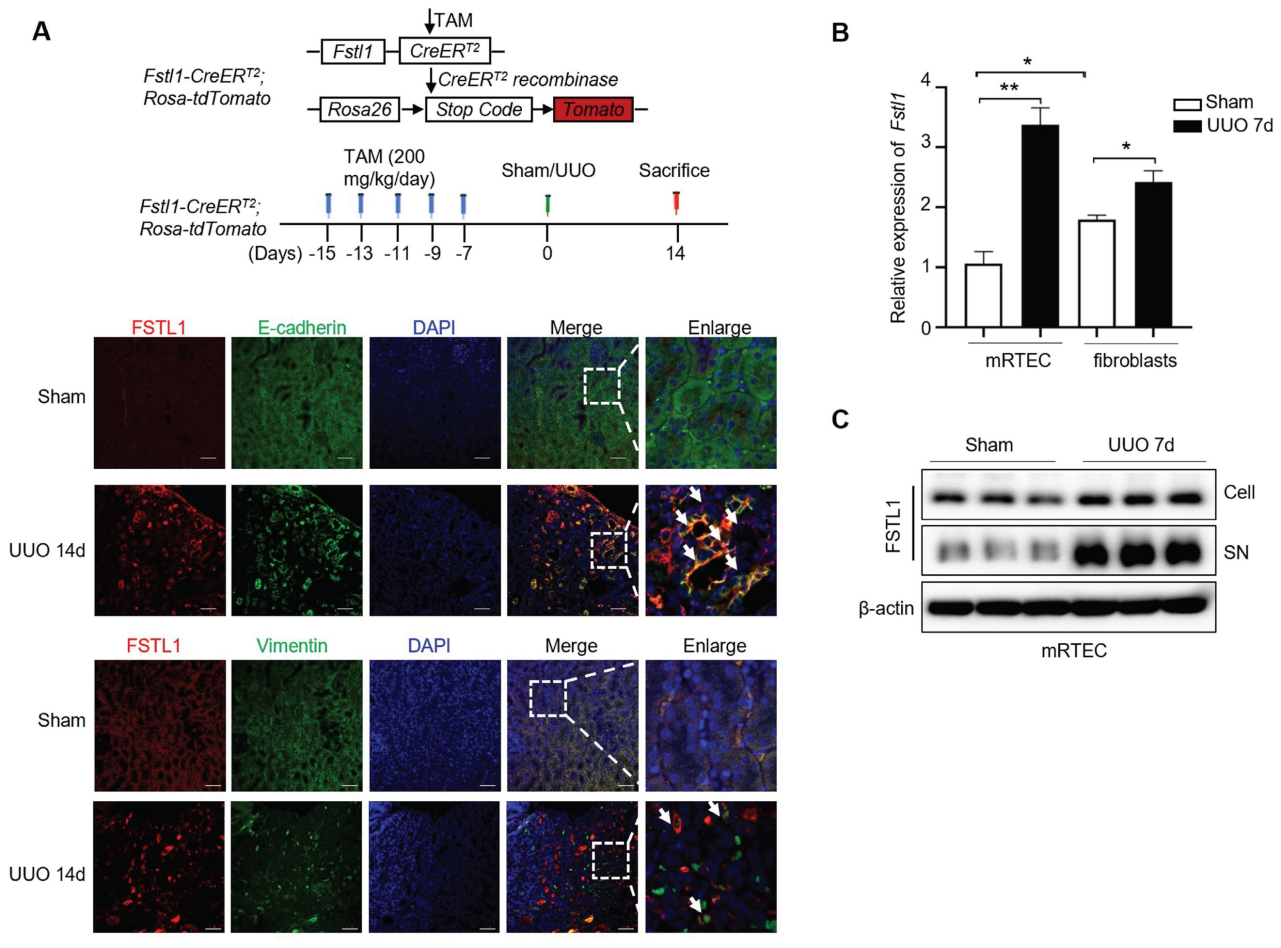
**Increased FSTL1 mainly derives from renal tubular epithelium of obstructed mouse kidney.** *Fstl1* lineage tracing mice (*Fstl1*-CreER^T2^; Rosa-tdTomato) were received daily intraperitoneal tamoxifen (TAM) injections for five consecutive days, followed by UUO treatment for 14 days. These kidneys were harvested for further analysis. The kidneys were then harvested for subsequent analysis. (A) Immunofluorescence analysis was performed to determine the co-localization of FSTL1 (red) and E-cadherin (green) or Vimentin (green) in the obstructed kidneys of *Fstl1* lineage tracing mice, with co-localization being indicated by yellow coloring. (B) qRT-PCR analysis was performed to measure the mRNA levels of *Fstl1* in the both primary mouse renal tubular epithelial cells (mRTEC) and interstitial fibroblasts, isolated from sham-operated and obstructed kidney tissues 7 days after UUO (n = 3 per group). The quantification of results was normalized to β-actin*.* Error bars represent the mean ± SEM. (C) Western blot analysis was performed to measure the protein levels of Fstl1 in both cellular extracts and culture medium (supernatant, SN) collected from isolated primary mRTECs derived from sham-operated and obstructed kidney tissues 7 days after UUO (n = 3 per group).

Previous studies have reported that FSTL1 expression is limited to the collecting ducts and nascent nephron epithelia during kidney organogenesis 31, and to the loop of Henle during adult life 26. However, the pathological expression pattern of FSTL1 in renal injury model remains controversial 25-27. As shown in Figure 1D, we found that FSTL1 was extensively expressed in the obstructed kidney tissue, but mostly expressed in the tubular epithelium. Moreover, a genetic *Fstl1* lineage tracing mouse (*Fstl1-creER^T2^; Rosa-tdTomato*) was employed to confirm increased FSTL1 production mainly derived from renal tubular epithelium cells. As shown in Figure 4A, FSTL1 (red) was highly co-localized with the epithelial marker E-cadherin (green) in kidneys subjected to UUO, but not with mesenchymal marker Vimentin (green). In addition, we isolated primary mouse renal tubular epithelial cells (mRTECs) and interstitial fibroblasts from kidney tissues 7 days post-UUO. Under physiological conditions, the mRNA expression of *Fstl1* was 1.8-fold higher in primary renal interstitial fibroblasts than that in mRTECs (Figure 4B). However, following UUO surgery, *Fstl1* mRNA expression was 1.4-fold higher in mRTECs than that in primary renal interstitial fibroblasts (Figure 4B). Furthermore, we observed that *Fstl1* mRNA expression was elevated by 3.0-fold in UUO-treated mRTECs and by 1.4-fold in UUO-treated primary renal interstitial fibroblasts, each compared with sham-operated controls (Figure 4B). In addition, western blot analyses also showed a significant increase of FSTL1 protein level in both cellular extracts and supernatant collected from isolated primary mRTECs following UUO treatment (Figure 4C). These data collectively demonstrate that increased FSTL1 is mainly derives from renal tubular epithelium of the obstructed mouse kidney.

### FSTL1 attenuates TNF-α-induced activation of NF-κB, release of IL-1β and IL-6 in HK2 cells

**Figure 5:**
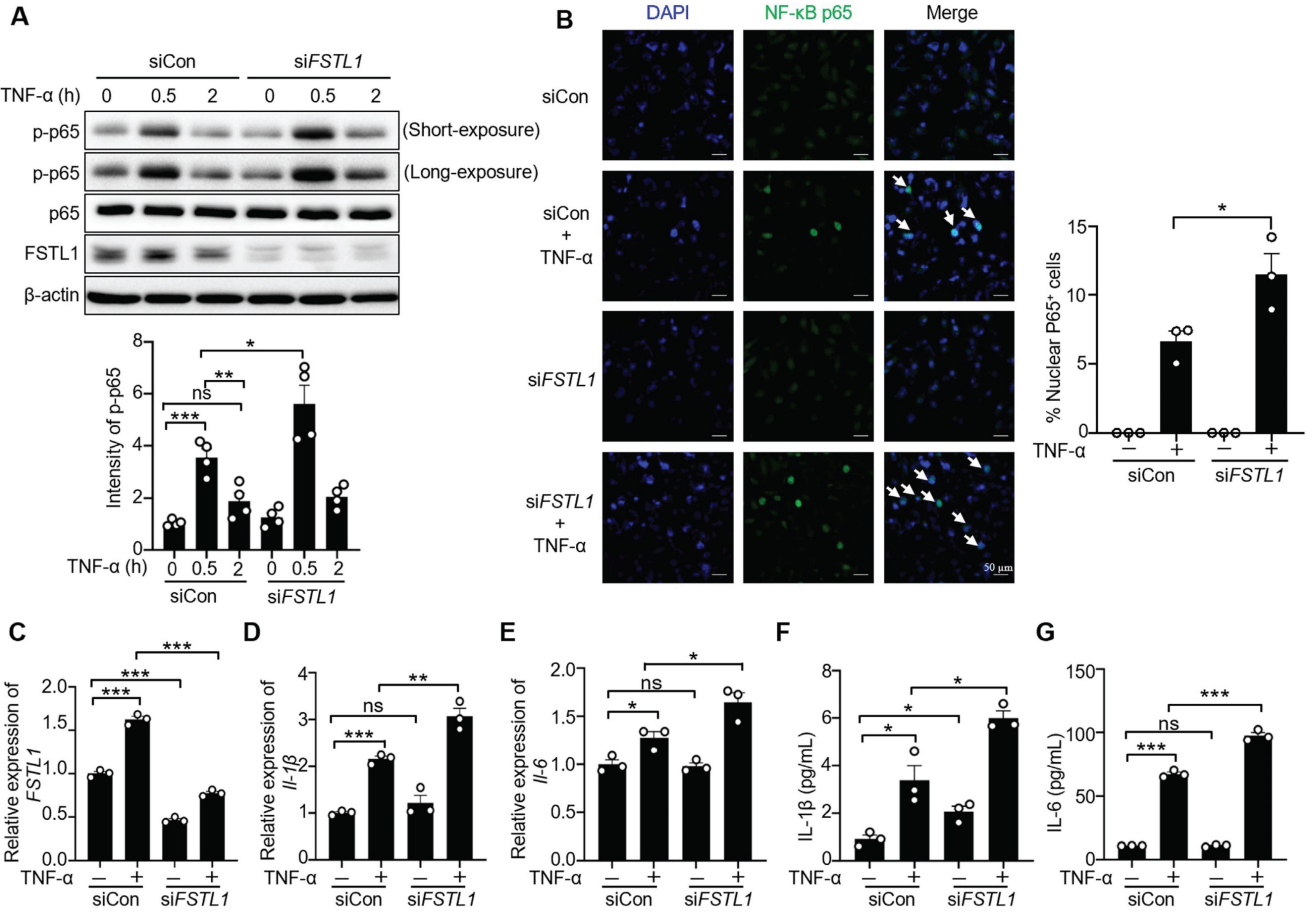
**Knockdown of *FSTL1* enhances TNF-α-induced phosphorylation and nuclear translocation of NF-κB P65 (p-p65), and release of IL-1β and IL-6 in HK2 human kidney epithelial cells.** (A) HK2 cells were transfected with control siRNA (siCon) or si*FSTL1* for three days, followed by stimulation with TNF-α (10 ng/mL) for 0, 0.5, and 2 hours. Subsequent immunoblotting of cell lysates was performed for p-p65, p65, FSTL1, and β-actin, with p-p65 intensity normalized to p65. (B) Following transfection with siCon or si*FSTL1* for three days, HK2 cells treated with TNF-α (10 ng/mL) for 0.5 hours were immunostained with an antibody against NF-κB P65 (green), and nuclei were stained with DAPI (blue). White arrows highlight the translocation of NF-κB P65 subunits into the nucleus, and the percentage of nuclear P65-positive cells was quantified. (C-G) HK2 cells transfected with siCon or si*FSTL1* for three days, and subsequently treated with TNF-α (10 ng/mL) for 16 hours, were analyzed for *FSTL1, IL-1β* and *IL-6* mRNA levels via qRT-PCR (C-E), with normalization to Gapdh. The supernatants were collected to assess IL-1β and IL-6 secretion using an ELISA kit (F and G). (A-F) The experiments were performed in triplicate, with error bars representing the mean ±SEM, and statistical significance denoted by *P < 0.05, **P < 0.01, ***P < 0.001; ns, not significant. Error bars represent the mean ± SEM.

**Figure 6:**
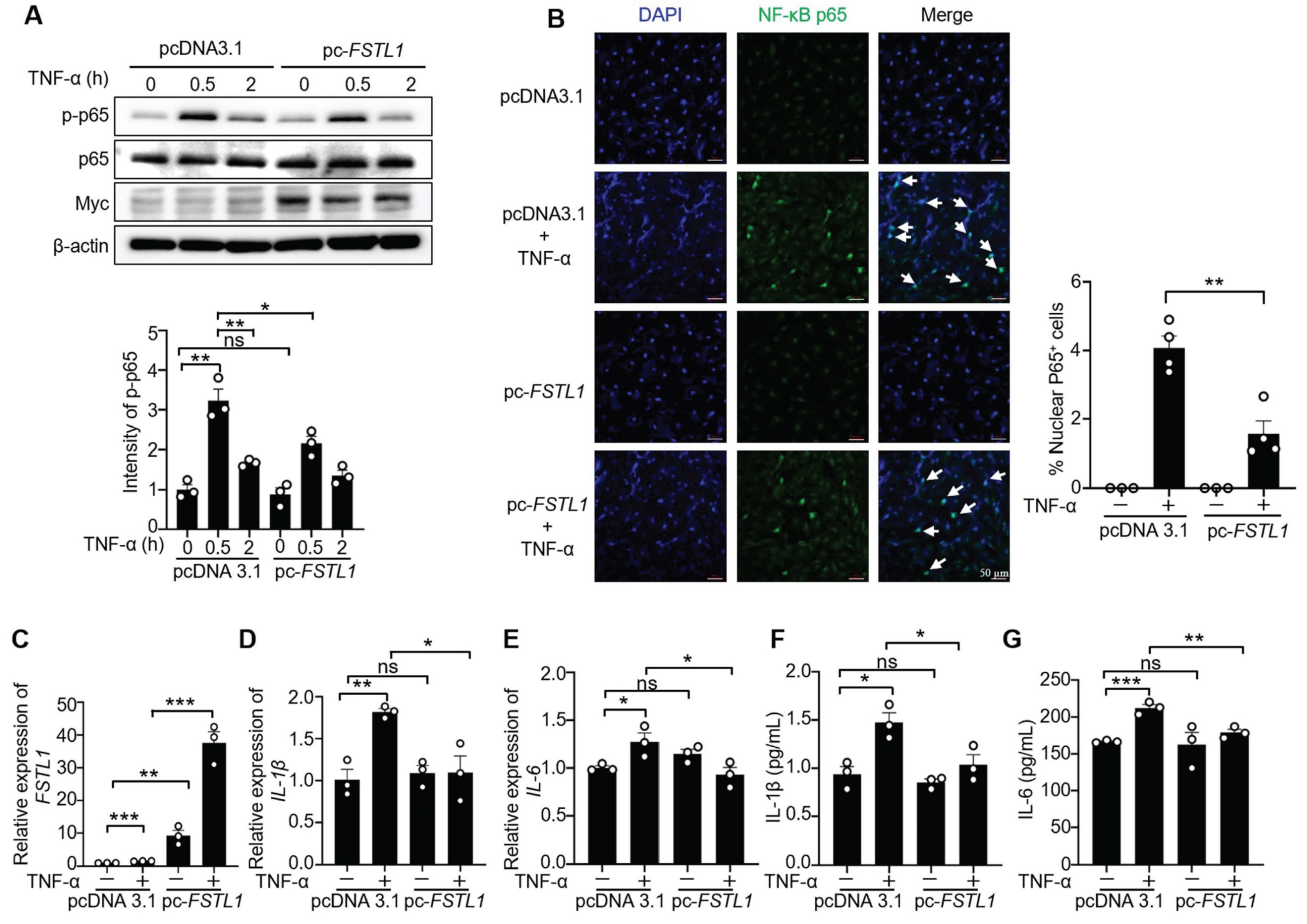
** Overexpression of *FSTL1* reduces TNF-α-induced phosphorylation and nuclear translocation of NF-κB P65 (p-p65), and release of IL-1β and IL-6 in HK2 human kidney epithelial cells.** (A) HK2 cells were transfected with the empty vector pcDNA 3.1 or pc-*FSTL1* plasmids for two days, followed by stimulation with TNF-α (10 ng/mL) for 0, 0.5, and 2 hours. Subsequent immunoblotting of cell lysates was performed for p-p65, p65, FSTL1, and β-actin, with p-p65 intensity normalized to p65. (B) Following transfection with pcDNA 3.1 or pc-*FSTL1* plasmids for 48 hours, HK2 cells treated with TNF-α (10 ng/mL) for 0.5 hours were immunostained with an antibody against NF-κB P65 (green), and nuclei were stained with DAPI (blue). White arrows highlight the translocation of NF-κB P65 subunits into the nucleus, and the percentage of nuclear P65-positive cells was quantified. (C-G) HK2 cells transfected with pcDNA 3.1 or pc-*FSTL1* plasmids for 48 hours, and subsequently treated with TNF-α (10 ng/mL) for 16 hours, were analyzed for *FSTL1*, *IL-1β* and *IL-6* mRNA levels via qRT-PCR (C-E), with normalization to Gapdh. The supernatants were collected to assess IL-1β and IL-6 secretion using an ELISA kit (F and G). (A-G) The experiments were performed in triplicate, with error bars representing the mean ±SEM, and statistical significance denoted by *P < 0.05, **P < 0.01, ***P < 0.001; ns, not significant.

Previous studies have shown that different cell type-derived FSTL1 exerts contrasting effects on inflammation^15^, ^23^, ^26^, ^29^. Here, we first examined the effect of FSTL1 on TNF-α induced NF-κB inflammatory pathway in HK2 kidney epithelial cells. We found that knockdown of *FSTL1* with* FSTL1* siRNA markedly enhanced TNF-α-induced NF-κB p65 phosphorylation (Figure 5A), as well as NF-κB p65 subunit nuclear translocation (Figure 5B). However, overexpression of *FSTL1* with Myc-tagged *FSTL1* plasmid obviously attenuated TNF-α-induced NF-κB p65 phosphorylation (Figure 6A), as well as NF-κB p65 subunit nuclear translocation (Figure 6B). These data suggest that FSTL1 negatively regulates the NF-κB pathway, and FSTL1 may play an anti-inflammatory role in HK2 cells.

We next investigated the effect of FSTL1 on the production of inflammatory cytokines induced by TNF-α in HK2 cells. Knockdown of *FSTL1* with* FSTL1* siRNA obviously increased the mRNA levels of *IL-1β* and *IL-6* (Figure 5C-E), as well as their release (Figure 5F-G). However, overexpression of *FSTL1* with Myc-tagged *FSTL1* plasmid significantly decreased the mRNA levels of *IL-1β* and *IL-6* (Figure 6C-E), as well as their release (Figure 6F-G). These findings indicate that FSTL1 may mitigate epithelial inflammation *via* negatively regulating NF-κB pathway.

### FSTL1 attenuates TNF-α-induced ICAM1 expression in HK2 cells and subsequent leukocyte recruitment

**Figure 7:**
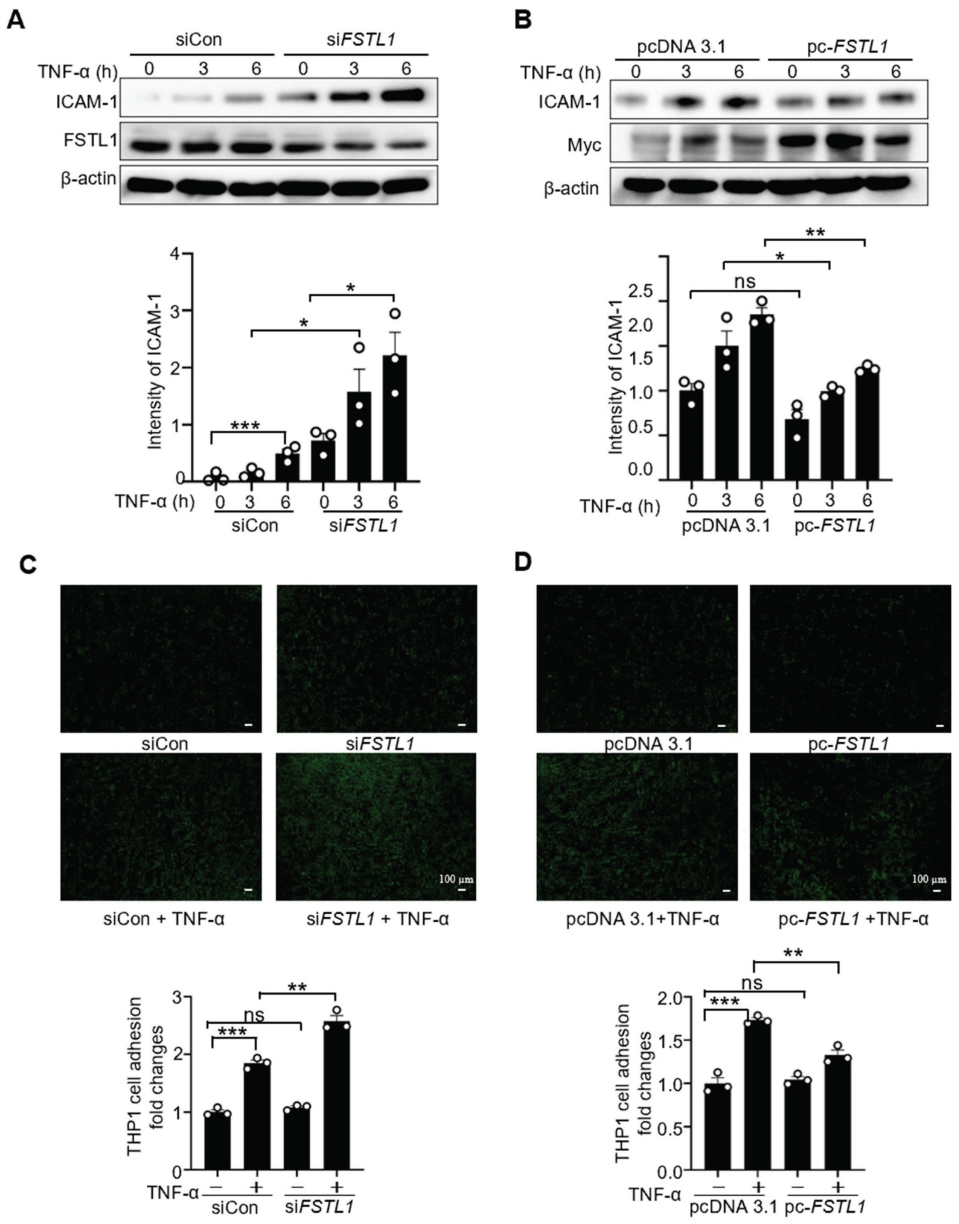
**FSTL1 reduces TNF-α-induced ICAM-1 expression, and monocyte adhesion to kidney epithelial cells.** (A-B) HK2 cells were transfected with either siCon or si*FSTL1* for three days (A), or with pcDNA 3.1 and pc-*FSTL1* plasmids for two days (B), followed by treatment with TNF-α (10 ng/mL) at 0, 3, and 6 hours. Subsequently, the cells were processed for western blot analysis to measure ICAM-1 expression levels, with normalization to β-actin. (C) Post-transfection with siCon or si*FSTL1* for three days, HK2 cells were treated with TNF-α (10 ng/mL) for 16 hours. Calcein AM-labeled THP-1 monocytes (5×10^5^) were added and co-incubated for one hour to evaluate monocyte adherence to epithelial cells, with the number of adherent Calcein AM-labeled THP-1 cells quantified. (D) HK2 cells transfected with the empty vector pcDNA 3.1 or pc-*FSTL1* plasmids for two days were treated with TNF-α (10 ng/mL) for 16 hours. Subsequent to the treatment, Calcein AM-labeled THP-1 monocytes (5×10^5^) were introduced and co-incubated for one hour, with monocyte adherence assessed through counting of the adherent Calcein AM-labeled THP-1 cells. (A-D) The experiments were performed in triplicate, and error bars represent the mean ±SEM, with statistical significance indicated by *P < 0.05, **P < 0.01, ***P < 0.001; ns, not significant.

ICAM-1 is a critical cell-surface glycoprotein facilitating the recruitment of leukocytes to inflamed epithelial tissues 11. Its expression is increased in endotoxin-treated epithelium via NF-κB activation 32. To investigate whether FSTL1 affects TNF-α-induced ICAM-1 expression, HK2 cells were transfected with either siCon or *siFSTL1* for three days, or with pcDNA 3.1 and pc*-FSTL1* plasmids for two days, followed by treatment with TNF-α at 0, 3, and 6 hours. Knockdown of FSTL1 markedly enhanced TNF-α-induced ICAM-1 expression (Figure 7A), whereas overexpression of *FSTL1* with Myc-tagged *FSTL1* plasmid significantly attenuated TNF-α-induced ICAM-1 expression in HK2 kidney epithelial cells (Figure 7B).

Next, we further determined whether FSTL1 affects leukocytes adhesion to the epithelium. THP-1 cells labeled with Calcein AM were then added to these HK2 cells, and their adhesive capacity was measured. Knockdown of FSTL1 with *FSTL1* siRNA enhanced TNF-α-induced THP-1 cell adhesion to HK2 cells (Figure 7C), whereas overexpression of FSTL1 reduced TNF-α-induced THP-1 cell adhesion to HK2 cells (Figure 7D). Taken together, these data suggest that FSTL1 may attenuate leukocyte recruitment through its negative role in reducing the NF-κB pathway, IL-1β and IL-6 release, and expression of ICAM-1.

### Systemic delivery of FSTL1 ameliorates renal inflammation and fibrosis after UUO

**Figure 8:**
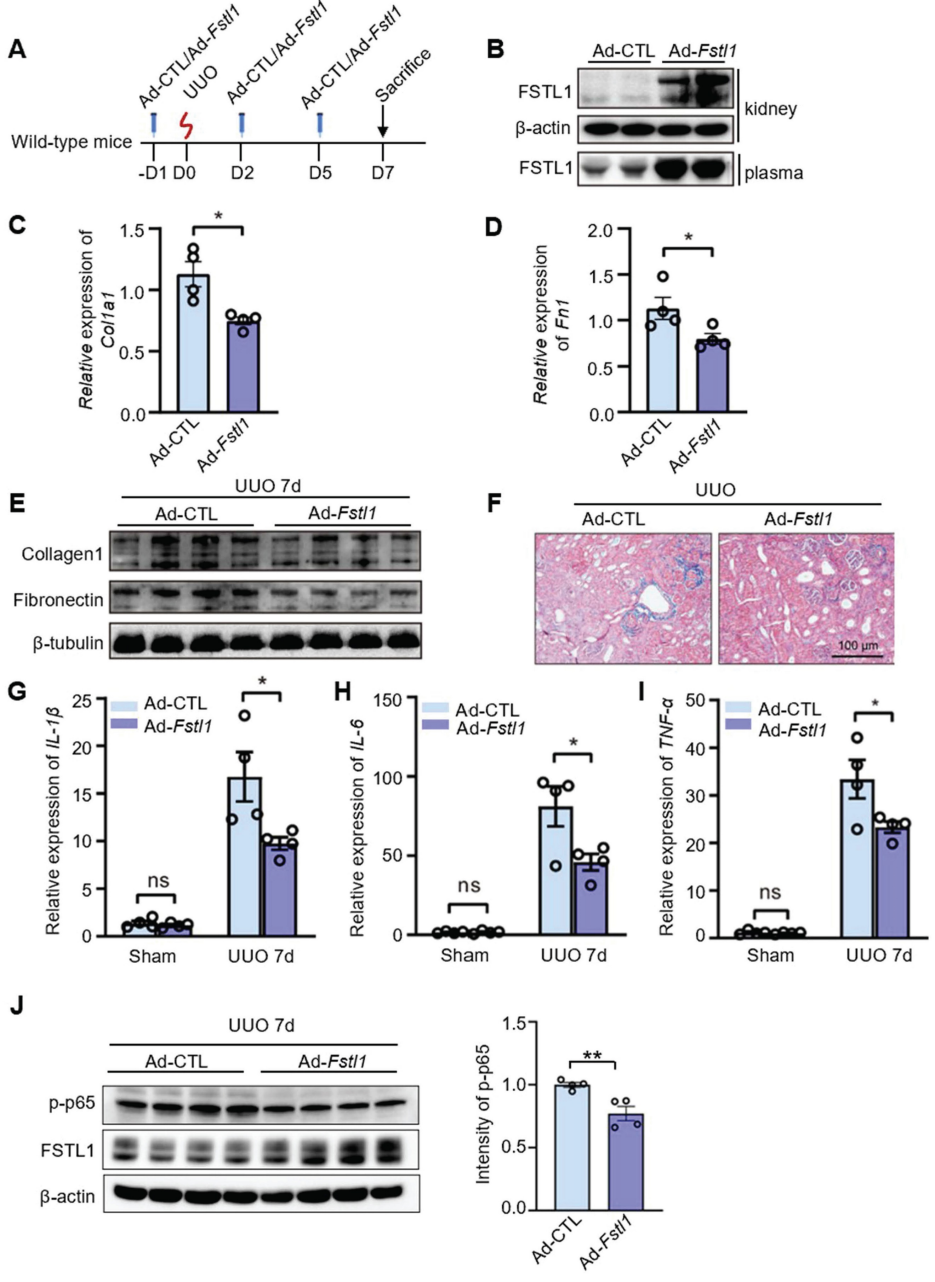
**Systemic administration of FSTL1 mitigates renal interstitial fibrosis and inflammation following UUO treatment.** (A) A dosing regimen using adenovirus-mediated gene delivery was employed in the UUO model. (B) Western blot analysis was conducted to determine FSTL1 levels in kidney tissues and plasma of wild-type mice two days after intravenous injection of control adenovirus (Ad-CTL, 1×10^9^ pfu) or *Fstl1*-expressing adenovirus (Ad*-Fstl1*, 1×10^9^ pfu). (C-E) qRT-PCR and western blot analyses were utilized to measure the expression levels of Collagen 1 and Fibronectin in obstructive kidney lysates from Ad-CTL and Ad*-Fstl1* treated mice. β-tubulin served as a loading control. (F) Masson's trichrome staining facilitated the histological evaluation of renal interstitial fibrosis. (G-I) The mRNA levels of *IL-1β*, *IL-6*, and *TNF-α* in sham and obstructive kidney lysates from Ad-CTL and Ad*-Fstl1* treated mice were quantified using qRT-PCR seven days post-UUO. (J) Western blot analysis was performed to measure the levels of p-p65 and FSTL1 in obstructed kidney lysates from mice treated with Ad-CTL or Ad-*Fstl1* after 7 days of UUO injury (n = 3 per group). The results in panels C, D, G, H, and I were normalized to Gapdh. (A-J) Error bars represent the mean ±SEM, with *P < 0.05 and **P < 0.01 indicating statistical significance.

To investigate the potential therapeutic effects of FSTL1 overexpression on renal injury, C57BL/6J mice were intravenously injected with an adenovirus encoding *Fstl1* (Ad-*Fstl1*) or a control adenoviral vector (Ad-CTL) 1 day prior to UUO and at 2 and 5 days after UUO treatment (Figure 8A). We first verified that Ad*-Fstl1* treatment resulted in significantly increased levels of FSTL1 in both kidney tissue and plasma (Figure 8B). Renal fibrosis was significantly ameliorated in mice treated with Ad*-Fstl1* compared with those receiving Ad-CTL, as indicated by the reduced mRNA and protein expression of major ECM components, such as collagen I and fibronectin (Figure 8C-E). The decreased collagen accumulation in Ad*-Fstl1*-treated mice was further supported by Masson's trichrome staining (Figure 8F). As expected, the mice treated with Ad*-Fstl1* showed an attenuated renal inflammation, as determined by mRNA levels of proinflammatory cytokines (*IL-1β*, *IL-6,* and *TNF-α)* in the obstructive kidney (Figure 8G-I). In addition, we also observed that the protein level of p-p65 in obstructed kidney lysates from mice treated with Ad*-Fstl1* was markedly lower than those treated with Ad-CTL (Figure 8J and Figure S3). Collectively, these results indicate that adenoviral-mediated overexpression of FSTL1 alleviates UUO-induced renal inflammation and subsequent fibrosis *in vivo*, suggesting that FSTL1 supplementation may represent a promising therapeutic approach for patients with progressive renal fibrotic diseases.

## Discussion

Renal fibrosis is commonly recognized as the ultimate pathway for nearly all chronic kidney disease (CKD) cases 5. The detailed molecular mechanisms driving CKD progression remain largely unknown. Currently, therapeutic alternatives are few. This study provides novel insights into the regulatory mechanisms of CKD and highlights the anti-fibrotic and anti-inflammatory properties of FSTL1 through experiments using a UUO mouse model. We present comprehensive data from animal studies, cellular analyses, and molecular investigations, along with key intervention evidence that supports FSTL1's potential as a therapeutic target for CKD.

Persistent inflammation is one of the most important initiators that promotes renal fibrosis 7, 9. UUO leads to an increase in immune cell infiltration and the expression of pro-inflammatory cytokines, thereby exacerbating fibrotic injury 33. Previous research has shown that FSTL1 negatively regulates the expression of inflammatory cytokines in the kidney following cisplatin treatment or subtotal nephrectomy 26, 27.

In line with these findings, our study demonstrated that the targeted deletion of *Fstl1* in mice exacerbated UUO-induced renal inflammation, whereas overexpressing FSTL1 via adenoviral transduction alleviated UUO-induced renal inflammation. Mechanistically, we identified that FSTL1 mitigated NF-κB-mediated secretion of inflammatory cytokines and leukocyte infiltration in human renal epithelial cells.

FSTL1, a well-conserved matricellular glycoprotein, is implicated in fibrosis across various organs 13. However, its role in fibrogenesis may be organ-specific and also dependent on its cellular origin. Our research has initially identified FSTL1 as a major pro-fibrotic factor in lung fibrosis. The administration of a neutralizing antibody against FSTL1 has been demonstrated to mitigate inflammation and subsequent fibrosis following lung injury induced by bleomycin, silica, or X-ray 20-22, 25. FSTL1 also exhibits pro-fibrotic effects in liver and skin fibrosis 24, 25, 34. Cardiac myocyte-derived FSTL1 serves cardioprotective functions in myocardial hypertrophy 15, but cardiac fibroblast-derived FSTL1 promotes cardiac fibroblast activation 23. However, the expression and function of FSTL1 in kidney fibrosis present some conflicting findings. A study using a subtotal nephrectomy model suggested that FSTL1 derived from cardiac myocytes can mitigate renal tubulointerstitial fibrosis 27. Conversely, research involving models of CKD with obstructed kidneys indicates that FSTL1 from fibroblasts promotes renal fibrogenesis 28, 29. In our current study, we have provided *in vivo* and* in vitro* evidence to ascertain the expression and derivation of FSTL1 in obstructed kidney tissues. We observed elevated FSTL1 expression in both CKD patients and a UUO-induced mouse model. Employing a genetic *Fstl1* lineage tracing mouse, we determined that increased FSTL1 primarily expressed in the renal tubular epithelium of obstructed kidneys. Additionally, FSTL1 protein levels were found to increase in mRTECs following UUO treatment. Moreover, our findings demonstrate the protective role of FSTL1 in renal fibrogenesis. Targeting deletion of *Fstl1 in vivo* exacerbated UUO-induced tubulointerstitial fibrosis, while gene transfer of *Fstl1* showed a mitigating effect. Based on these previous studies, we hypothesized that mesenchymal cell-derived FSTL1 plays a pro-fibrotic role in organ fibrosis. In this study, mRTECs are identified as the primary source of injury-induced FSTL1 expression following UUO surgery. Consequently, FSTL1 exerts a predominant anti-fibrotic effect in the progression of UUO-induced renal fibrosis.

Previous studies have shown that FSTL1 modulates several signaling pathways in kidney injury/fibrosis in a cell type-dependent manner. Hayakawa *et al.* showed that cardiac myocyte-derived FSTL1 promotes the AMP-activated protein kinase (AMPK) pathway to exert protective effects against renal injury 27. Chen *et al.* demonstrated that FSTL1 facilitates the formation of tight junctions in renal epithelia via Akt signaling pathway 35. Zhang *et al.* found that fibroblast-derived FSTL1 enhances Wnt/β-catenin signaling to promote kidney fibrosis 29. In our study, we found that obstructed kidneys subjected to 3, 7 or14 days of UUO exhibited a significantly increased mRNA expression of *β-catenin* compared with the sham-operated kidneys (Figure S2B). However, we failed to observe the significant difference in the mRNA levels of *β-catenin* between the obstructed kidneys of WT and *Fstl1^+/-^* mice (Figure S2B). Nevertheless, our current *in vivo* and *in vitro* studies demonstrate that FSTL1, derived from renal tubular epithelial cells, inhibits NF-κB-mediated signaling, ultimately mitigating UUO-induced renal fibrosis. In future studies, we will utilize cell-specific *Fstl1* knockout mice, combined with RNA sequencing, to further investigate these and other signaling pathways involved in the pathogenesis of renal fibrosis.

**Figure 9:**
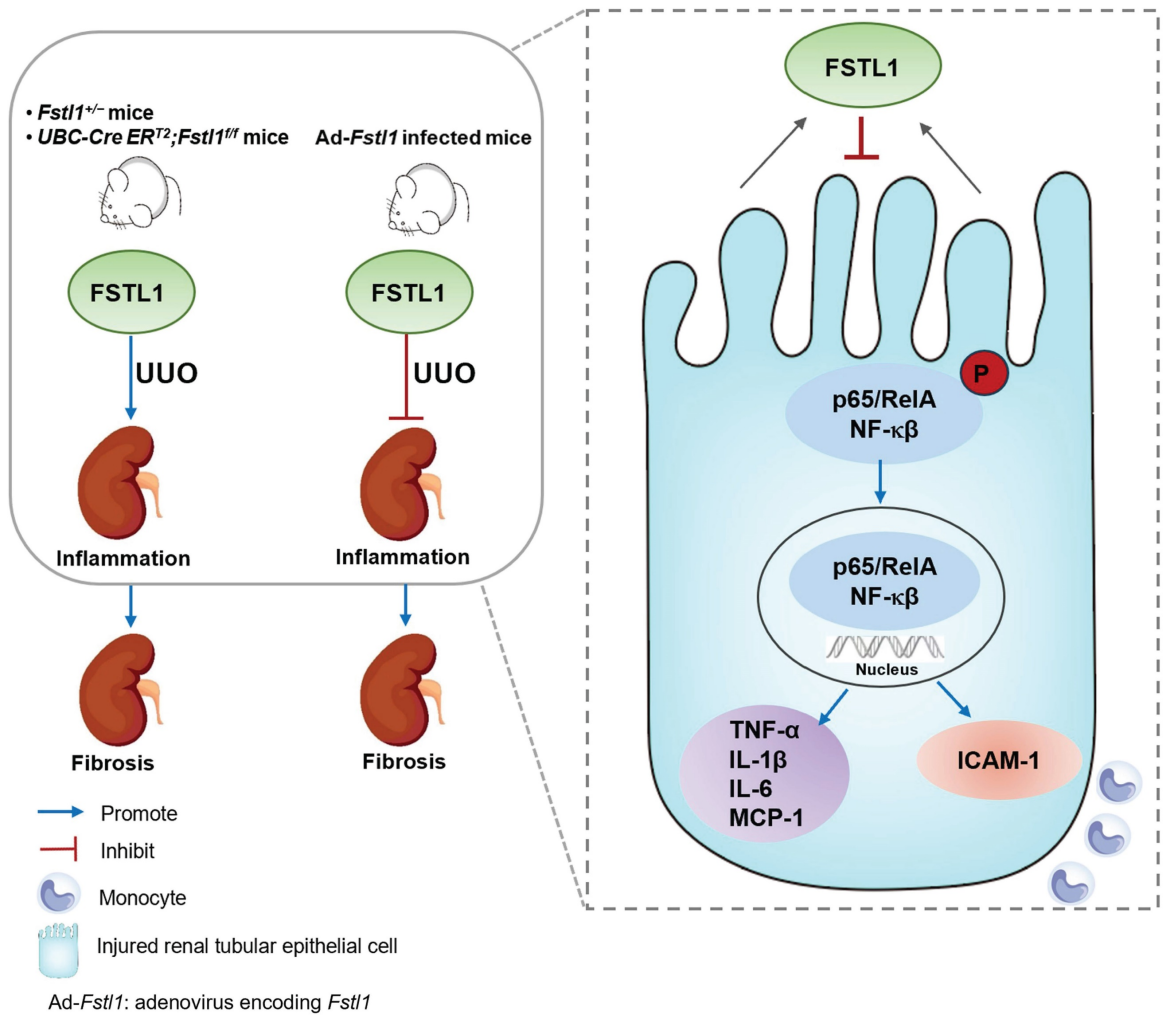
**Schematic summary of this study.** Our findings solidly confirmed the protective role of FSTL1 in kidney inflammation and fibrosis* in vivo*. Increased FSTL1 production is mainly from the renal tubular epithelium in the obstructed mouse kidney. FSTL1 mitigates renal fibrosis partly by suppressing renal epithelial inflammation through inhibiting NF-κB activation. The precise mechanisms of how FSTL1 derived from renal tubular epithelial cells in the UUO model affects activation and function of renal fibroblasts should be further explored.

In summary, this study showed that FSTL1 plays a protective role in UUO-induced renal inflammation and fibrosis, and targeted deletion of* Fstl1* exacerbated renal inflammation and fibrosis in response to kidney injury (Figure 9). We speculated that the mechanisms of this action partially depend on protection against renal epithelial inflammation, thereby reducing the inflammatory milieu and limiting subsequent fibrotic damage in the kidney. Our ongoing research is focused on elucidating the specific mechanisms by which FSTL1, derived from renal tubular epithelial cells in the UUO model, affects the activation and function of renal fibroblasts. This study will offer novel insights into understanding the pathogenesis of renal fibrosis and provide a novel therapeutic target for patients with CKD.

## Materials and Methods

### Animals

C57BL/6J mice at 8 weeks were obtained from Vital River Laboratories (Beijing, China). *Fstl1^+/-^* mice were bred following previously established protocols 20. We obtained *Fstl1-CreER^T2^, UBC-CreER^T2^* and *Fstl1^flox/flox^* mice from the Model Animal Research Center at Nanjing University (Nanjing, China). Ai14 (Rosa-tdTomato) reporter mice were sourced from the Jackson Laboratory. By crossing *Fstl1*-CreER^T2^ mice with Ai14 mice, we produced *Fstl1*-CreER^T2^; Ai14^+/+^ (also named as *Fstl1-CreER^T2^; Rosa-tdTomato*) mice 36, 37. Similarly, *UBC-CreER^T2^* mice were crossed with *Fstl1^+/-^* mice to create *UBC-CreER^T2^;Fstl1^+/-^* progeny. These F1 heterozygous offspring were subsequently crossed with *Fstl1^flox/flox^* mice, resulting in an F2 generation with four distinct genotypes, including* Ubc-CreER^T2^;Fstl1^flox/-^* transgenics and control *Fstl1^flox/+^* mice for further experiments. These mice above were allowed free access to water and food. UUO surgery was conducted in accordance with previously described methods 38, involving ligation of the left ureter with 4-0 silk under general anesthesia to create an obstruction. In contrast, sham-operated mice underwent only ureter exposure without ligation. Mice used in the UUO study were euthanized at specific time points for analysis. All animal experiments were approved by the Nankai University Animal Care and Use Committee (approval no. 2024-SYDWLL-000637).

### Expression profile dataset and analysis

The dataset for gene profiling of CKD kidneys, specifically examining *FSTL1* expression, was sourced from previous studies 30. The raw set of gene array data is available in the NCBI Gene Expression Omnibus (GEO accession: GSE66494).

### Fstl1 recombinant adenovirus construction

The adenovirus used to express mouse FSTL1 (NM_008047, Ad-*Fstl1*) was obtained from GeneChem (Shanghai, China). To achieve* in vivo* overexpression of FSTL1, mice received an injection of 1×10^9^ plaque-forming units (pfu) per mouse of Ad-*Fstl1* via the tail vein. A control adenovirus (Ad-CTL), administered at the same dosage, was used as the negative control in this study.

### Plasmid and siRNA transfection

Human *FSTL1* cDNA (NM_007085.5) was inserted into the pcDNA3.1/myc-His (-) A vector (Invitrogen, Carlsbad, CA, USA). The* FSTL1* gene was amplified using specific primers: the forward primer, 5'-AAGCTTATGTGGAAACGCTGGCTC-3', and the reverse primer, 5'-TCTAGAGATCTCTTTGGTGCTCACTC-3'. HK2 cells underwent transfection with the constructed pc-*FSTL1* or with a pcDNA3.1 control plasmid utilizing Lipofectamine 2000, in accordance with the instructions provided by the manufacturer. Additionally, small interfering RNAs (siRNAs) targeting human FSTL1 were introduced into HK2 cells using the GeneMute siRNA transfection reagent.

### RNA extraction and qRT-PCR analysis

RNA extraction and qRT-PCR were executed using established protocols 39. Total RNA was isolated from kidney tissues or renal tubular epithelial cells using TRIzol reagent (Invitrogen, Carlsbad, CA, USA). cDNA was synthesized with the YEASEN reverse transcription kit (11142ES10, YEASEN, China). mRNA quantification was conducted utilizing SYBR Green Master Mix (11184ES03, YEASEN, China), with normalization against mouse β-actin or human GAPDH. The primer sequences used are provided in **Table S1**.

### Western blot analysis

Frozen kidney samples were processed by homogenizing them in 1x RIPA buffer, which included both proteinase and phosphatase inhibitors (Sigma-Aldrich, USA). Protein concentrations were detected with the BCA assay kit (YH372890, Thermo Fisher Scientific, USA). The proteins were then separated using SDS-PAGE and transferred onto PVDF membranes. After blocking, the membranes were probed with specific primary antibodies overnight at 4°C. Subsequently, they were incubated with HRP-conjugated secondary antibodies at room temperature for 2 h. Protein bands were detected using an enhanced ECL kit (Thermo Scientific Pierce, USA). Details on the primary antibodies used can be found in **Table S2**.

### Histology and immunohistochemistry

Kidney tissues were initially fixed in 10% neutral buffered formalin for 24 hours, followed by dehydration, paraffin embedding, and sectioning into 5 μm slices. The sections were then stained using H&E, Sirius Red, or Masson's trichrome, according to protocols provided by Sigma-Aldrich. For immunohistochemistry (IHC), sections were first deparaffinized using xylene and alcohol, then submerged in citrate buffer. Antigen retrieval was facilitated through high-pressure heating. Subsequently, the sections were cooled and blocked with 5% normal goat serum. Primary antibodies targeting Fstl1 and α-SMA were applied overnight at 4°C. This was followed by a 15-minute incubation with HRP-polymer secondary antibodies. The detection was performed using a MaxVision HRP-Polymer anti-Mouse/Rabbit IHC Kit (Kit-5010/5020/5030, MXB Biotechnologies). Quantitative analysis of Sirius Red and α-SMA staining was carried out by capturing random cortical images (10 fields per kidney) from each mouse and calculating the percentage of positively stained areas in each microscopic field.

### Immunofluorescence staining

HK2 cells were grown to 50 - 60% confluence in glass-bottom dishes and treated with TNF-α (10 ng/mL) for 30 minutes. After treatment, the cells were rinsed with PBS and fixed using 4% paraformaldehyde for 40 minutes. Then, the cells were blocked with 5% BSA in TBST for 40 minutes. Overnight incubation at 4°C with a p65 primary antibody was performed, followed by application of an Alexa Fluor-488-conjugated secondary antibody (S11223, Invitrogen, Carlsbad, CA, USA) to facilitate immunofluorescence visualization. Nuclei staining was done using DAPI (Santa Cruz Biotechnology, USA). Imaging was conducted using a Zeiss Axio Imager LSM710 microscope, and analysis was completed with Zeiss software.

### Hydroxyproline assay

Kidney tissues were processed, weighed, and analyzed for hydroxyproline content using a hydroxyproline assay kit from Nanjing Jiancheng Bioengineering Institute, following the protocol specified by the manufacturer.

### ELISA assay

The concentrations of IL-1β and IL-6 in the cell supernatant were measured using commercial ELISA kits, following the procedures outlined by the manufacturers. Details about these ELISA kits can be found in **Table S3**.

### Assay of THP-1 adherence to renal epithelial cells

Human kidney epithelial cells (HK2) were subjected to siRNA transfection for three days or plasmid transfection for two days, followed by a 16-hour treatment with TNF-α (10 ng/mL). Concurrently, THP-1 cells, a human monocytic leukemia cell line, were stained with Calcein AM at 7.5 μM for 40 minutes at 37°C in a 5% CO2 atmosphere. Labeled THP-1 cells (5×10^5^ cells), were then added to the HK2 cells in each well and co-incubated for one hour under the same conditions. Post co-incubation, the wells were rinsed with pre-warmed RPMI medium for removing non-adherent THP-1 cells. The absorbance of the adherent cells was read on a microplate reader at excitation/emission of 485/530 nm. Absolute cell counts were determined by comparing these fluorescence readings with those from a dilution series of Calcein AM-tagged THP-1 cells suspended in RPMI medium. This experimental procedure was performed in triplicate to ensure accuracy and repeatability.

### Statistical analyses

All data are reported as the mean ± SEM. Statistical analyses were performed using Prism version 8.4.3. Differences between two groups were assessed by an unpaired Student's t-test. For comparisons involving multiple groups, one way ANOVA was used. *P* < 0.05 was deemed statistically significant.

## Supplementary Material

Supplementary figures and tables.

## Abbreviations

FSTL1: Follistatin-like 1
CKD: chronic kidney diseases
UUO: unilateral ureteral obstruction
HK2 cells: human kidney epithelial cells
ECM: excessive extracellular matrix
ICAM-1: intercellular adhesion molecule 1
THP-1 cells: a human monocytic leukemia cell line
FN: fibronectin
mRTECs: mouse renal tubular epithelial cells
