# Supplementary material for: Renal tubular epithelial-derived follistatin-like 1 protects against UUO-induced renal fibrosis in mice via inhibiting NF-κB-mediated epithelial inflammation
Source: Theranostics. 2025 Jan 20;15(6):2413–27. doi: 10.7150/thno.100969 (PMC11840719; doi:10.7150/thno.100969)
Supplement: Supplementary file 1 — Supplementary figures and tables. [file thnov15p2413s1.pdf]

Supplementary Figures

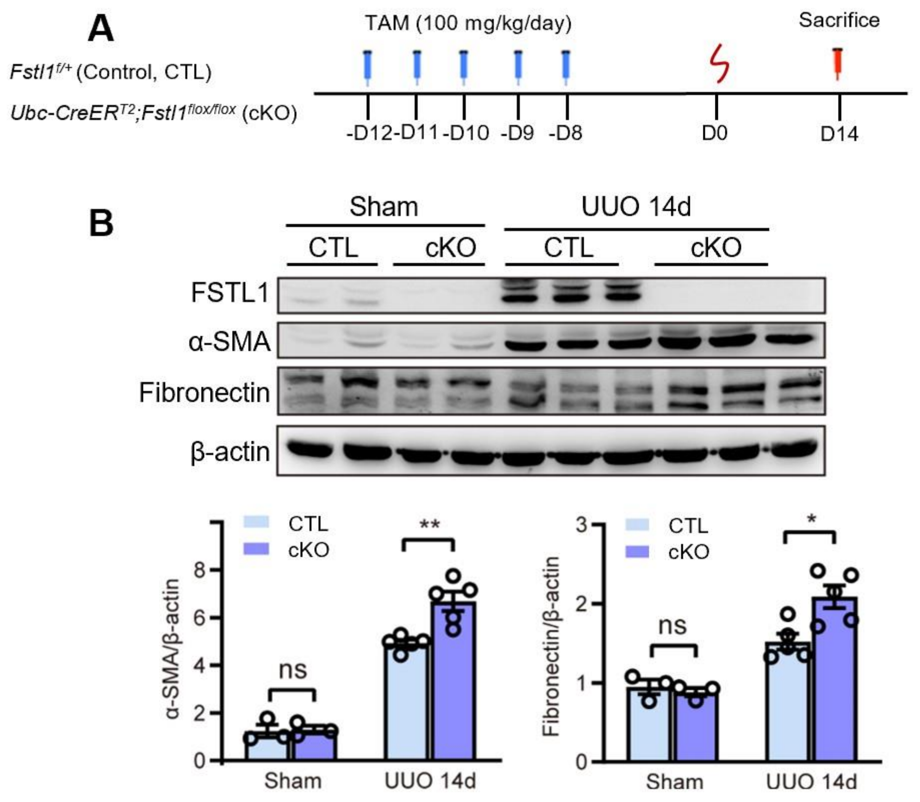

Figure S1. *Fstl1* conditional knock-out exacerbates renal interstitial fibrosis following UUO. (A) Schematic representation shows that *Ubc-CreER*^{T2}*;Fstl1*$^{flox/flox}$ (cKO) transgenic mice and the control *Fstl1*$^{flox/+}$ (CTL) mice, received daily intraperitoneal injections of tamoxifen for five days, followed by UUO surgery seven days later. (B) Western blot analysis provided a comparative representation of Fstl1, α-SMA, and Fibronectin protein expression in the kidney lysates from CTL and cKO mice at 14 days post-UUO. β-actin served as a loading control. Error bars represent the mean ± SEM, with statistical significance denoted by *P < 0.05 and **P < 0.01; ns, not significant.

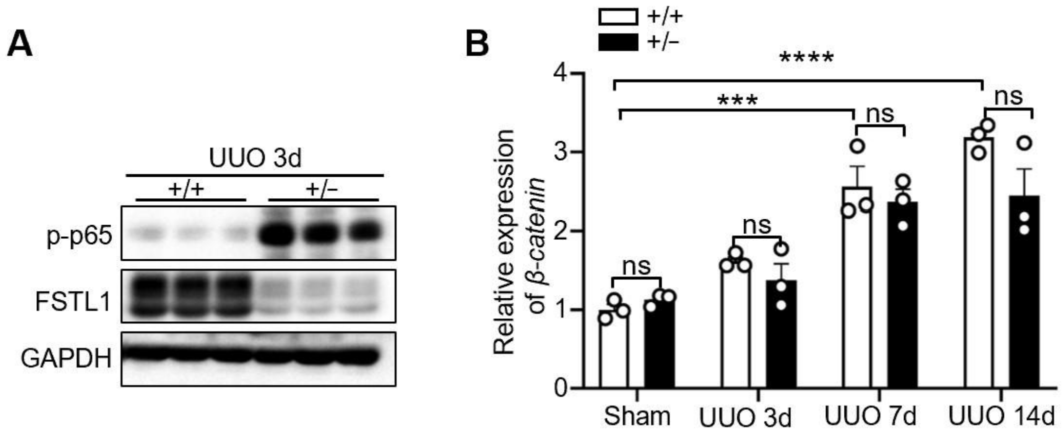

Figure S2. *Fstl1* haplodeficiency promoted the UUO-induced activation of NF-κB signaling in kidney tissues. (A) Western blot analysis was performed to assess the protein levels of p-p65 and FSTL1 in both sham-operated and obstructed kidney lysates from wild-type and *Fstl1*$^{+/-}$ mice after 3 days of UUO (n = 3 per group). (B) qRT-PCR analysis was performed to measure the mRNA level of *β-catenin* in both sham-operated and obstructed kidney lysates from wild-type and *Fstl1*$^{+/-}$ mice after 3, 7, 14 days of UUO (n = 3 per group). ***P < 0.001; ****P < 0.0001; ns, not significant.

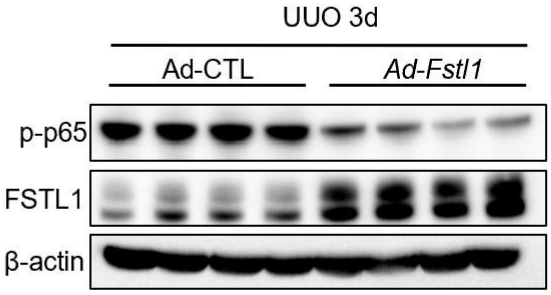

Figure S3. Systemic administration of FSTL1 attenuates the UUO-induced activation of NF-κB signaling in kidney tissues. Western blot analysis was performed to measure the protein levels of p-p65 and FSTL1 in obstructed kidney lysates from mice treated with Ad-CTL or Ad-*Fstl1* after 3 days of UUO injury (n = 3 per group).

Table 1.
Primers for qRT-PCR

Gene		Sequence 5'---3'
**M Fstl1**	Forward	TTATGATGGGCACTGCAAAGAA
NM_008047.5	Reverse	ACTGCCTTTAGAGAACCAGCC
**M IL-1β**	Forward	GAAATGCCACCTTTTGACAGTG
NM_008361.4	Reverse	TGGATGCTCTCATCAGGACAG
**M IL-6**	Forward	TAGTCCTTCCTACCCCAATTTCC
NM_031168.2	Reverse	TTGGTCCTTAGCCACTCCTTC
**M TNF-α**	Forward	CCCTCACACTCAGATCATCTTCT
NM_008361.4	Reverse	GCTACGACGTGGGCTACAG
**M Acta2**	Forward	GCTGGTGATGATGCTCCCA
NM_007392.2	Reverse	GCCCATTCCAACCATTACTCC
**M Col1a1**	Forward	CCAAGAAGACATCCCTGAAGTCA
NM_007742.3	Reverse	TGCACGTCATCGCACACA
**M Fn1**	Forward	GTGTAGCACAACTTCCAATTACGAA
NM_010233	Reverse	GGAATTTCCGCCTCGAGTCT
**M MCP-1**	Forward	AACTACAGCTTCTTTGGGACA
NM_011333.3	Reverse	CATCCACGTGTTGGCTCA
**M β-actin**	Forward	AGGCCAACCGTGAAAAGATG
NM_007393.3	Reverse	AGAGCATAGCCCTCGTAGATGG
**H GAPDH**	Forward	GGAGCGAGATCCCTCCAAAAT
NM_008084.3	Reverse	GGCTGTTGTCATACTTCTCATGG
**H FSTL1**	Forward	TCTGTGCCAATGTGTTTTGTGG
NM_007085.5	Reverse	TGAGGTAGGTCTTGCCATTACTG
**H IL-1β**	Forward	ATGATGGCTTATTACAGTGGCAA
NM_000576.3	Reverse	GTCGGAGATTCGTAGCTGGA
**H IL-6**	Forward	ACTCACCTCTTCAGAACGAATTG
NM_000600.5	Reverse	CCATCTTTGGAAGGTTCAGGTTG

Abbreviations: M: mouse; H: human; Fn1: Fibronectin; Cola1: Collagen type I

Table 2.
Primary Antibodies

Antibody	Cat No.	Manufacturer	Source of species
FSTL1	AF1738	R&D	Goat
Human FSTL1	AF1694	R&D	Goat
Collagen type I	Ab21286	Abcam	Rabbit
Fibronectin	AB268020	Abcam	Rabbit
α-SMA	SC-32251	Santa Cruz	Mouse
E-cadherin	610181	BD	Mouse
β-actin	AC026	ABclonal	Rabbit
β-tubulin	AC021	ABclonal	Mouse
p-NF-κB p65	93H1	Cell Signaling	Rabbit
NF-κB p65	66535-1-Ig	proteintech	Mouse
ICAM-1	4915s	Cell Signaling	Rabbit

Table 3
ELISA kits information

Parameter	Cat No.	Manufacturer	Species
IL-1β	88-7261-88	Invitrogen	Human
IL-6	88-7066-88	Invitrogen	Human
